# Study on the value of Inhibin B in the diagnosis of nasopharyngeal carcinoma and its correlation with traditional Chinese medicine syndromes: An observational study

**DOI:** 10.1097/MD.0000000000038416

**Published:** 2024-06-07

**Authors:** Nuoya Ma, Xin Deng, Qing Liu, Fei Xu, Qi Guo, Kun Yan, Yan Yang, Guoying Zou

**Affiliations:** a Department of Medical Laboratory Science, Clinical Medical School, Hunan University of Traditional Chinese Medicine, Changsha, Hunan, China; b Department of Clinical Laboratory, Brain Hospital of Hunan Province the Second People's Hospital of Hunan Province, Changsha, Hunan, China.

**Keywords:** INHA, INHBB, Inhibin B, nasopharyngeal carcinoma, TCM syndromes

## Abstract

To investigate the expression of Inhibin B between various clinical stages, Chinese medicine dialectic typing, and in nasopharyngeal carcinoma (NPC) tissues and serum, and to evaluate the potential of Inhibin B as a new biomarker for NPC. Paraffin specimens of pathologically confirmed NPC tissues and paracancerous tissues were retrospectively collected, and the expression of Inhibin α (INHA) and Inhibin βB (INHBB) was detected by SP method, and their relationship with clinicopathological indexes was analyzed; in addition, patients with NPC who had received radiotherapy were included as the study subjects, and Epstein–Barr virus DNA (EBV-DNA), INHA, and INHBB in patients were detected by using the fluorescence quantitative polymerase chain reaction, enzyme-linked immunosorbent assay, and chemiluminescent immuno-sandwiching method, respectively. EBV-DNA, EBV-viral capsid antigen-immunoglobulin A (VCA IgA), INHA, and INHBB were detected in the patients, respectively, and their relationships with traditional Chinese medicine (TCM) patterns were also analyzed. The expression of INHA and INHBB in NPC tissues was lower than that in paracancerous tissues, and the expression of INHA in NPC patients was correlated with lymphatic metastasis, clinical staging, and TCM staging; the levels of EBV-DNA and VCA IgA were higher than that of healthy populations in NPC patients and were higher than that of patients with stage III + IV than that of patients with stage I + II, and the levels of INHA and INHBB were lower than those of healthy populations and were lower than those of patients with stage III + IV than that of patients with stage I + II. The levels of INHA and INHBB in nasopharyngeal cancer patients were lower than those in healthy people, and the levels in stage III + IV patients were lower than those in stage I + II patients. The levels of EBV-DNA and VCA IgA in nasopharyngeal cancer patients were correlated with the Chinese medicine patterns, and had different patterns. The expression of Inhibin B may be related to the progression of NPC, and it has certain typing significance for different TCM syndromes of NPC, which is helpful for TCM typing diagnosis.

## 1. Introduction

Nasopharyngeal carcinoma (NPC) is a malignant tumor with highly metastatic characteristics that occur at the top and side walls of the nasopharyngeal cavity. It is a malignant tumor with unique biological characteristics, mostly occurring in southern China, with obvious regional aggregation, ethnic susceptibility, and a high family incidence.^[1]^ Most patients with nasopharyngeal carcinoma are diagnosed with clinical stage III or IV disease, and the 5-year survival rate is 50% to 60%.^[2]^ In ancient Chinese medicine, there was no description about the name of nasopharyngeal carcinoma, but it was divided into such categories as “cervical carcinoma with cachexia,” “stony lumbar carbuncle,” and “nasosinusitis” according to different descriptions of clinical symptoms.^[3]^ Therefore, modern doctors often treat it according to its clinical manifestations, such as epistaxis, nasal congestion, runny nose, tinnitus and ear swelling, headache, and lymphadenopathy.^[4]^ There is no unified standard for syndrome differentiation and classification of nasopharyngeal carcinoma at present. “Traditional Chinese medicine (TCM) Syndrome Differentiation and Diagnosis” divides nasopharyngeal carcinoma into 4 syndrome types: Phlegm-type condensation, Blood-type condensation, Burning sleepy knot type, and Empty stagnate poisonously.^[5]^ At present, circulating Epstein–Barr virus DNA (EBV-DNA) load in plasma has been identified as a tumor marker of nasopharyngeal carcinoma, and approximately 53% to 96% of patients with nasopharyngeal carcinoma can detect the presence of EBV-DNA in blood before treatment, which is regarded as an important indicator for screening, monitoring, and predicting the progress of nasopharyngeal carcinoma.^[6]^ However, there is currently no uniform standard for the detection of EBV-DNA in different centers. A meta-analysis showed that the sensitivity of plasma EBV-DNA in the diagnosis of nasopharyngeal carcinoma is only 76% (95% CI: 68–83%) and the specificity is 93% (90–96%),^[7]^ which may not be enough to predict the prognosis of patients with nasopharyngeal carcinoma. IgA antibodies to Epstein–Barr virus nucleocapsid antigen (VCA) reflect the mucosal immune response to the pathogen, and in the case of nasopharyngeal carcinoma, mucosal epithelial carcinoma IgA-EBV reflects the role of EBV in the pathogenesis of nasopharyngeal carcinoma.^[8]^ However, because the detection operation is cumbersome, requires certain clinical experience, is highly subjective, and lacks precision and accuracy, it can interfere with serological diagnosis or even misdiagnosis as a single indicator.^[9]^ Therefore, it is necessary to identify more specific, sensitive, and easily detectable markers to assist in the early diagnosis and treatment of nasopharyngeal carcinoma. Inhibin is a member of the transforming growth factor-β (TGF-β) superfamily and is a heterodimer consisting of a common α subunit bound to either the βA subunit (Inhibin A) or the βB subunit (Inhibin β).^[10]^ TGF-β family is involved in the malignant progression of tumors, and INHA (Inhibin α), as one of the target genes of this family, plays a role in slowing the progression of cancer in some cases.^[11]^ In one study, homologous recombination in mouse embryonic stem cells was found to cause INHA target deletion, leading to gonadal mesenchymal tumors in mice. Thus, INHA is considered to be a key negative regulator affecting the proliferation of gonadal stromal cells, as well as a factor with tumor-suppressor activity.^[12]^ INHBB (Inhibin βB) can regulate the production of follicles and negatively regulate follicle-stimulating hormone (FSH),^[13]^ and is, therefore, the best independent marker for the follow-up of adult granulosa cell tumors.^[14]^ Most previous studies on INHBB have focused on the reproductive system,^[15]^ and in recent years, INHBB has been recognized as a valuable biomarker in a variety of cancer types.^[16]^ It has been found that knockdown of INHBB expression in oral squamous cell carcinoma promotes metastasis and accelerates the invasion of cancer cells.^[17]^ Currently, studies on the relationship between nasopharyngeal carcinoma diagnosis and Inhibin B have not been reported, and a previous study by our group revealed that INHBB inhibited the loss of apoptosis resistance and migration of NPC cells through the TGF-β signaling pathway at both the cellular and protein levels, which in turn affected the invasion and metastasis of nasopharyngeal carcinoma,^[18]^ therefore, the present study intends to further validate the basic research in clinical diagnostics and treatment, utilizing immunohistochemistry; therefore, the present study is intended to further validate the basic research in clinical diagnosis and treatment by using immunohistochemistry and serology to detect the expression of INHA and INHBB in cancer tissues and serum, and to explore their relationship with clinical stage and TCM patterns, so as to provide a more clinical basis for the combination of traditional Chinese and Western medicine in the diagnosis and treatment of nasopharyngeal carcinoma.

## 2. Materials and methods

### 2.1. Study design and classification of nasopharyngeal carcinoma patients

From September 2016 to March 2020, 35 paraffin specimens diagnosed as nasopharyngeal carcinoma were collected, and the corresponding adjacent tissues were used as the normal control group. Blood samples were collected from 134 patients with nasopharyngeal carcinoma between May 2021 and December 2022, and the control group was normal. The staging standard of nasopharyngeal carcinoma refers to the 8th edition TNM staging of nasopharyngeal carcinoma published by the Union for International Cancer Control/United States Joint Commission on Cancer (UICC/AJCC) in 2017. The inclusion criteria were as follows:

Head and neck magnetic resonance imaging (MRI), nasopharyngoscopy, chest computed tomography (CT), abdominal ultrasound examination, and bone scan clearly diagnosed nasopharyngeal carcinoma, and the pathological diagnosis was nonkeratinized cancer or nonkeratinized undifferentiated cancer, all of which excluded reproductive system diseases and other malignant diseases. Without other antitumor treatments, the paraffin-embedded specimens of patients were intact.

The dialectical classification of TCM syndromes is performed by senior TCM doctors with reference to the differential diagnosis of TCM syndrome. There are 4 syndrome types, namely, Phlegm-type condensation (nasal congestion and bloody nose, headache and weightlessness, fullness in the ear, or excessive phlegm, chest tightness, tiredness and lethargy, nausea and anorexia, reddish tongue, fat or toothed tongue, white or greasy tongue coating, and slippery pulse) and Blood-type condensation (stuffy in the ear, tinnitus and deafness, bloody nose, expectoration of a small amount, reddish tongue, white or yellowish fur, thin or slow pulse) and the Burning sleepy knot (severe headache, snot and blood, foul-smelling breath, cough with thick phlegm, heavy tinnitus and deafness, heartburn and insomnia, bitter mouth and dry throat, drowning and yellow bowel movement, red tongue, yellow moss or yellowish greasy moss, slippery or number of strings in the pulse), Empty stagnate poisonously (nasal congestion with snot and blood, tinnitus and deafness, accompanied by headache and dizziness, thinness and weakness, or night sweating, heartburn and heat, lumbar and knee soreness and tenderness, red tongue with little moss and a thin pulse).

This study was approved by the Ethics Committee of Hunan Brain Hospital (The second People’s Hospital of Hunan Province). All participants have informed consent.

### 2.2. Immunohistochemical analysis

The expressions of INHA and INHBB were detected using the immunohistochemical SP method. The paraffin specimens were cut to a thickness of 2.5 μm and baked in an oven at 72 °C for 30 minutes, deparaffinized, and placed in 10 mM citrate buffer for antigen repair. Endogenous peroxidase was removed with phosphate buffer solution (PBS). The primary antibody was incubated with 1% bovine serum albumin as a diluent, and the INHA and INHBB antibodies (Abcam, Boston) were diluted to a concentration of approximately 1:250 and left to stand overnight in a refrigerator at 4 °C. Instead of the primary antibody, PBS was used as a negative control. The primary antibody was rinsed with PBS 3 times and then incubated with the secondary antibody at room temperature for 1 hour. The secondary antibody was washed with PBS 3 times, and then restained with hematoxylin staining solution after the addition of drops of diaminobenzidine (DAB) colorant (Maxim Crop., Fujian, China), dehydrated, and blocked with gum.

All sections were read by 2 senior pathologists using a double-blind method, and the positive signals were localized in the cytoplasm, which was scored according to the strength of the positive signal cytoplasm with a yellowish, tan, or brown background, and according to the strength of staining of the positive signals of the INHA and INHBB, and the number of positive cells was scored in a composite manner.

### 2.3. Real-time fluorescence quantitative polymerase chain reaction (PCR) analysis

Two milliliters of venous blood from nasopharyngeal cancer patients and healthy adults were collected in EDTA-K2 sterile test tubes for examination, collect 1 mL of whole blood was collected in dry glass test tubes, and equal amounts of saline were added and mixed gently. Add 500 μL of lymphocyte isolation solution into the dry glass test tube, add the diluted whole blood into the glass test tube with lymphocyte isolation solution by pipette slowly, centrifuge at low speed for 20 minutes, aspirate the intermediate layer of leukocytes, centrifuge for 5 minutes, removed the supernatant, and then add 50 μL of DNA extract into the centrifugal precipitate, mixed well, processed at 100 °C for 10 minutes, and centrifuge at 12,000 rpm for 5 minutes. Take 40 μL/person of EBV PCR solution (Shengxiang Corp., Hunan, China), add 3 μL/person of Taq enzyme, mix well, dispense 43 μL/tube into 0.2 mL centrifuge tubes, and set aside. Add 2 μL of the supernatant of the treated samples into the centrifuge tube, centrifuge at 8000 rpm for several seconds, and the sample tank was placed in the instrument (Thermo Fisher Scientific, Waltham). Samples were amplified at 93 °C for 2 minutes, 93 °C for 45 seconds, 55 °C for 60 seconds, and 10 cycles of 93 °C for 30 seconds, 55 °C for 45 seconds, 30 cycles.

The results were interpreted as positive if the amplification curve of the test sample showed an S-shaped curve and the EBV-DNA assay value was >4.00E + 02 copies/mL.

### 2.4. Enzyme-linked immunosorbent assay (ELISA)

An ELISA was used to detect serum EBV-VCA IgA. One hundred microliters of sample diluent was added to the microtiter plate, and 10 μL of the sample to be examined was added to the microtiter plate. One well was set up as the negative and positive controls, and the liquid was removed from the wells after incubation for 30 minutes. Three hundred microliters of washing solution were added to each well, and the plate was left to stand for 30 seconds. Liquid was discarded, the plate was patted dry, and the above procedure was repeated 4 times; 100 μL of enzyme conjugate was added to each well. After incubation for 20 minutes, wash the plate, add 50 μL of substrate solution A and B to each well, incubate at 37 °C for 10 minutes, add 50 μL termination solution, and mix gently. Set the enzyme labeling instrument at dual-wavelength 450 nm/630 nm, measure the value of A in each well, and read the value within 30 minutes.

### 2.5. Chemiluminescent immunoassay analysis

After centrifuging the specimen, the serum was pipetted into a reaction cup and loaded into the tester-dedicated sample holder. The iFlash3000H Chemiluminescent Immunoassay Analyzer (YHLO Corp., Shenzhen, China) was used to perform the test, the test reagents were selected, calibration was performed, and the test was started after calibration.

### 2.6. Statistical methods

SPSS 19.0 software was used for statistical analysis. Quantitative data that approximately obeyed a normal distribution were expressed as mean ± standard deviation, while those that did not follow a normal distribution were expressed as median (interquartile spacing), and qualitative data were expressed as percentages. The statistics of age data were analyzed by ANOVA, and the statistics of sex data were analyzed using the chi-square test. For differences in the expression of INHα and INHβB in different tissues, the paired chi-square test was used, with a test level of α = 0.05. Overall comparisons of the blood indices in different clinical stages and TCM syndromes were performed using the Kruskal–Wallis test, with a test level of α = 0.05. Multiple comparisons were performed using the Mann–Whitney test with the Bonferroni method to correct for multiple comparisons. The Bonferroni method was used to correct the test level α′ = 0.0167 and α′ = 0.0083. EBV-DNA, EBV-VCA IgA, and INHA were analyzed by the receiver operating characteristic (ROC) curve and logistic regression. INHBB in nasopharyngeal carcinoma, the diagnostic value of EBV-DNA, EBV-VCA IgA, INHA, and INHBB in nasopharyngeal carcinoma were analyzed using the ROC curve and logistic regression. The area under the curve (AUC), sensitivity, and specificity were calculated, and the optimal cutoff value was determined using the Youden index (YI). The AUC area of 0.7 to 0.9 indicated a good diagnostic value.

## 3. Results

### 3.1. Expression of INHA and INHBB in nasopharyngeal carcinoma tissues and paracancerous tissues

**Figure 1.**
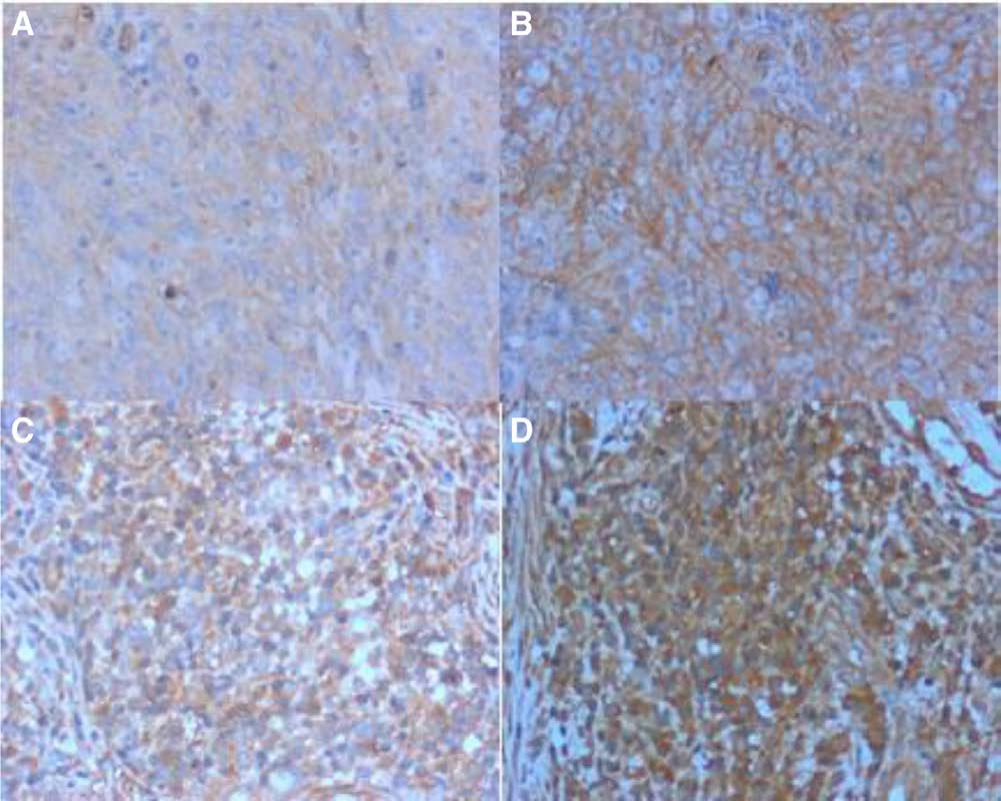
Expression of inhibin α (INHA) in nasopharyngeal carcinoma and paracancerous tissues detected by immunohistochemistry (×200). (A) Low expression of INHA in nasopharyngeal carcinoma tissues; (B) high expression of INHA in nasopharyngeal carcinoma tissues; (C) low expression of INHA in nasopharyngeal carcinoma paracancerous tissues; (D) high expression of INHA in nasopharyngeal carcinoma paracancerous tissues.

**Figure 2.**
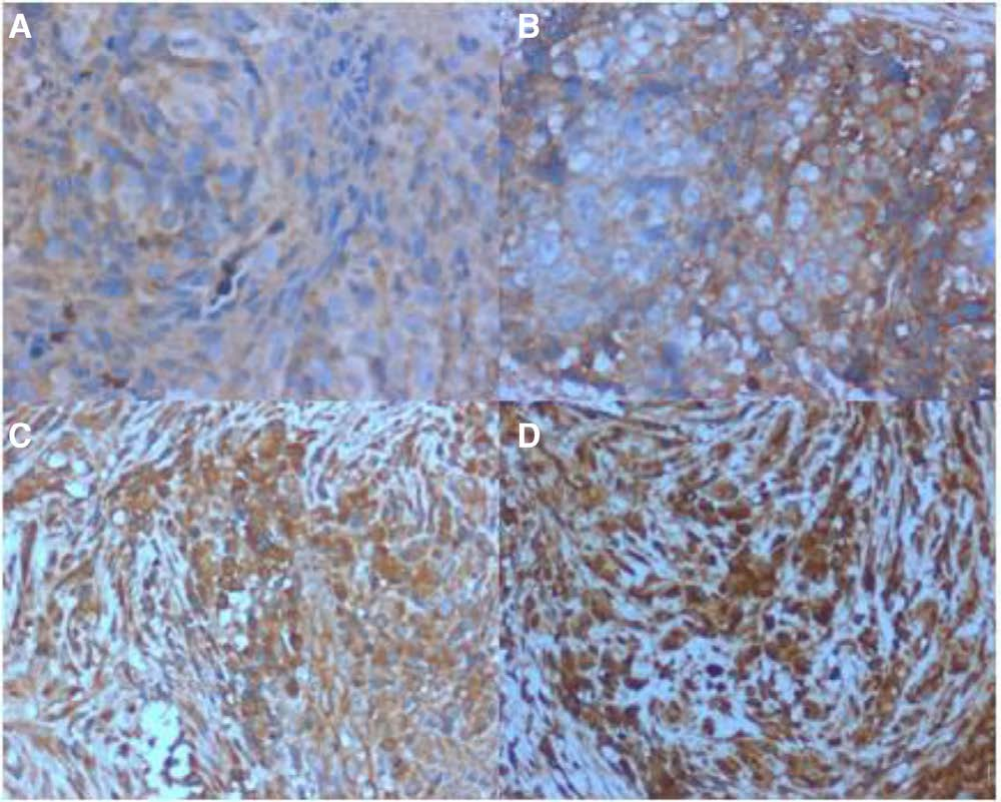
Expression of inhibin βB (INHBB) in nasopharyngeal carcinoma and paracancerous tissues detected by immunohistochemistry (×200). (A) Low expression of INHBB in nasopharyngeal carcinoma tissues; (B) high expression of INHBB in nasopharyngeal carcinoma tissues; (C) low expression of INHBB in nasopharyngeal carcinoma paracancerous tissues; (D) high expression of INHBB in nasopharyngeal carcinoma paracancerous tissues.

The positive staining of INHA and INHBB was mainly localized in the cytoplasm, and the positive signal intensity cytoplasm showed a background ranging from light yellow to brown to dark brown (Figs. 1 and 2). The immunohistochemical results are presented in Table 1. The positive expression rates of INHA and INHBB in 35 nasopharyngeal carcinoma tissues were 17.14% (6/35) and 34.29% (12/35), and in paracancerous tissue, the positive expression rates were 88.57% (31/35) and 68.57% (24/35), respectively. The differences in the expression of INHA and INHBB in nasopharyngeal carcinoma tissues and paracancerous tissues were statistically significant (*P* < .05). The results showed that the expression of both INHA and INHBB in nasopharyngeal carcinoma tissues were lower than that in paracancerous tissues (Table 1).

**Table 1:** Expression of INHA and INHBB in nasopharyngeal carcinoma tissues and paracancerous tissues.

	INHA		χ^2^	*P*	INHBB		χ^2^	*P*
	Positive	Negative			Positive	Negative		
Cancerous tissue	6	29	35.831	.000	12	23	8.235	.004
Paraneoplastic tissue	31	4			24	11		

### 3.2. Relationship between INHA and INHBB expression and clinicopathologic indexes in nasopharyngeal carcinoma patients

The expression of INHA and INHBB in patients with NPC was related to clinical stage, lymphatic metastasis, and TCM syndromes (*P* < .05), and the positive rates of INHA and INHBB expression in patients with stages III and IV were lower than that in stages I and II, and the positive rate of INHA and INHBB expression in patients with lymphatic metastasis was also significantly lower. In addition, the expression of INHA and INHBB was not related to patients’ sex, age, and T stage, and the difference was not statistically significant (*P* > .05) (Table 2).

**Table 2:** Expression of INHA and INHBB in nasopharyngeal carcinoma tissues and paracancerous tissues detected by immunohistochemical method.

Clinical parameters	Number of examples	INHA expression intensity		*P*	INHBB expression intensity		*P*
		High expression	Low expression		High expression	Low expression	
Sex							
Male	23	3	20	.391	7	16	1.000
Female	12	3	9		4	8	
Age							
≤60 yr old	26	4	22	.635	7	19	.416
>60 yr	9	2	7		4	5	
Clinical stage							
I + II	7	4	3	.009	5	2	.021
III + IV	28	2	26		6	22	
T staging							
T1 + T2	11	3	8	.352	5	6	.451
T3 + T4	24	3	21		7	17	
Lymphatic transfer							
Transferred	30	2	28	.001	8	22	.038
Not transferred	5	4	1		4	1	
TCM syndromes							
Blood-type condensation	3	1	2	.016	2	1	.031
Phlegm-type condensation	8	4	4		5	3	
Empty stagnate poisonously	12	0	12		1	11	
Burning sleepy knot	12	1	11		3	9	

### 3.3. Comparison of serologic test results between nasopharyngeal carcinoma patients with different clinical stages and the healthy group

According to the clinical staging criteria, there were 35 cases of stages I and II and 99 cases of stages III and IV. Compared with the 32 cases in the healthy group, the differences in the levels of EBV-DNA, VCA IgA, INHA, and INHBB in patients with different clinical stages were statistically significant (*P* < .001). The levels of EBV-DNA and VCA IgA were higher in nasopharyngeal cancer patients than in the healthy population, and they were higher in stage III + IV patients than in stage I + II patients (*P* < .0167). The levels of INHA and INHBB in nasopharyngeal cancer patients were significantly lower than those in the healthy population, and those in stage III + IV patients were lower than those in stage I + II patients (*P* < .0167) (Table 3).

**Table 3:** Comparison of serologic test results between healthy population and patients with different clinical stages.

Group	n	EBV-DNA (copies/mL)	VCA IgA (s/co)	INHA (pg/mL)	INHBB (pg/mL)
Healthy group	32	238.53 ± 58.27	0.51 ± 0.29	60.79 ± 20.77	64.55 ± 21.48
I + II stage	35	3.25E2 (5.40E1)*	0.95 (1.05)*	3.14 (3.56)*	42.30 (22.40)*
III + IV stage	99	4.43e6 (5.00E7)*#	1.60 (1.29)*#	0.50 (0.97)*#	35.20 (17.20)*#
χ^2^		106.316	44.201	110.539	39.026
*P*		*<*.001	*<*.001	*<*.001	*<*.001

* *P* < α*′* = 0.0167 compared to the healthy group.

#*P* < α*′* = 0.0167 compared with stage I + II.

### 3.4. Comparison of serologic test results of nasopharyngeal carcinoma patients with different TCM evidence types

According to the TCM syndromes, there were 37 cases of patients with burning sleepy knot, 38 cases of patients with empty stagnate poisonously, 27 cases of patients with Phlegm-type condensation, and 32 cases of patients with Blood-type condensation. There was no statistically significant difference in the levels of EBV-DNA and VCA IgA between different Chinese medicine diagnosis types (*P* > .05), but there was a statistically significant difference in the levels of INHA and INHBB among patients with different Chinese medicine diagnosis types (*P* < .05). Comparison within groups showed that INHA levels were higher in patients with Phlegm-type condensation than in patients with empty stagnate, and the difference was statistically significant (*P* = .003). The INHBB levels of patients with deficiency and empty stagnate poisoning, Phlegm-type condensation, and Blood-type condensation were all significantly higher than those of the burning sleepy knot (*P* < .0083) (Table 4).

**Table 4:** Comparison of serologic test results of nasopharyngeal cancer patients with different TCM types.

	n	EBV-DNA (copies/mL)	VCA IgA (s/co)	INHA (pg/mL)	INHBB (pg/mL)
Burning sleepy knot	37	5.86E5 (8.11E6)	1.41 (1.35)	1.04 (1.13)	28.20 (17.40)
Empty stagnate poisonously	38	2.55E6 (5.94E7)	1.51 (1.42)	0.50 (1.63)	40.15 (12.60)*
Phlegm-type condensation	27	4.80E4 (5.85E6)	1.52 (1.64)	1.65 (4.00)*#	39.20 (21.90)*
Blood-type condensation	32	3.67E4 (5.72E6)	1.57 (1.51)	1.77 (3.00)#	39.75 (28.50)*
	χ^2^	6.120	0.588	11.618	13.563
	*P*	.106	.899	.009	.004

* *P <* αʺ = 0.0083 compared to Burning sleepy knot.

#*P* < αʺ = 0.0083 compared to Empty stagnate poisonously.

### 3.5. ROC curve analysis of each index and multiple indexes combined to diagnose nasopharyngeal carcinoma

**Figure 3.**
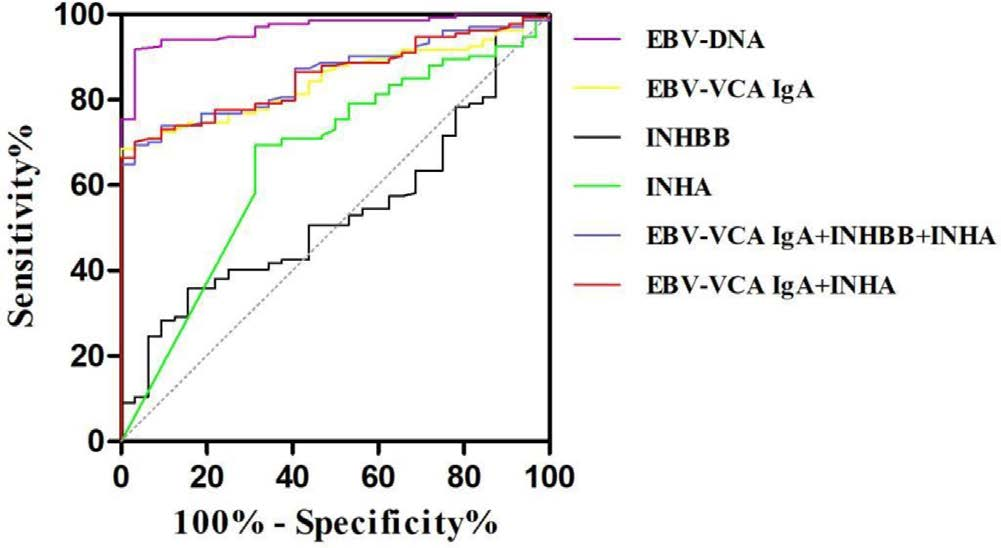
ROC curves of various markers and INHA and INHBB tests for the diagnosis of nasopharyngeal carcinoma. INHA = Inhibin α, INHBB = Inhibin βB, ROC = receiver operating characteristic curve.

The ROC curve analysis showed that the combination of EBV-VCA IgA, INHA, and INHBB could improve the diagnostic value of nasopharyngeal carcinoma, with an AUC of 0.857 (95% CI: 0.801–0.913). The optimal cutoff value for the combined diagnosis of nasopharyngeal carcinoma with EBV-VCA IgA, INHA, and INHBB was 0.838, and the Youden index was 0.663, which corresponded to a sensitivity of 69.4% and specificity of 96.9% (Fig. 3).

### 3.6. ROC curve analysis of Chinese medicine patterns of nasopharyngeal carcinoma diagnosed by each index and multiple indexes in combination

**Figure 4.**
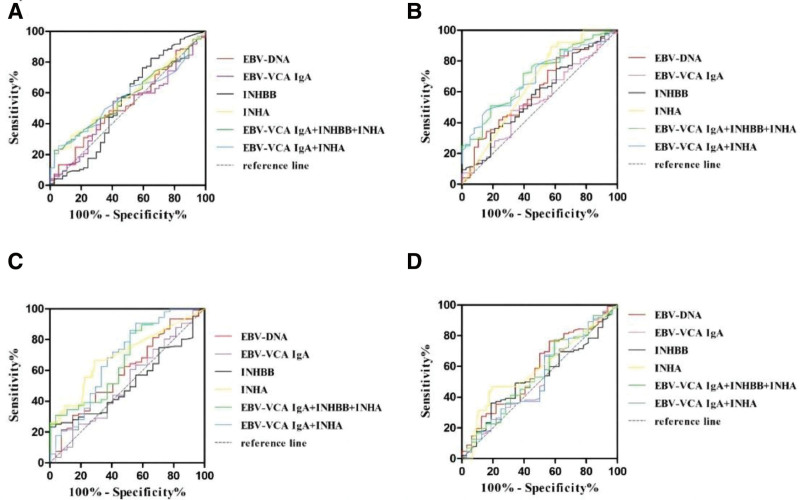
(A) ROC curve of Burning sleepy knot nasopharyngeal carcinoma. (B) ROC curve of Empty stagnate poisonously nasopharyngeal carcinoma. (C) ROC curve of Phlegm-type condensation nasopharyngeal carcinoma. (D) ROC curve of Blood-type condensation nasopharyngeal carcinoma. ROC = receiver operating characteristic curve.

ROC curve analysis showed that the AUC of EBV-DNA, EBV-VCA IgA, INHBB, INHA, EBV-VCA IgA + INHBB + INHA, EBV-VCA IgA + INHA for Burning sleepy knot nasopharyngeal carcinoma were 0.528, 0.509, 0.538, 0.579, 0.576, 0.568 and *P* > .05, so the above indexes had no diagnostic efficacy for Burning sleepy knot nasopharyngeal carcinoma. The AUC of EBV-DNA, EBV-VCA IgA, INHBB, INHA, EBV-VCA IgA + INHBB + INHA, EBV-VCA IgA + INHA of Empty stagnate poisonously nasopharyngeal carcinoma was 0.613, 0.542, 0.579, 0.655, 0.705, 0.691, EBV-DNA, INHA, EBV-VCA IgA + INHBB + INHA, and EBV-VCA IgA + INHA had AUC > 0.5 and *P* < .05, therefore, EBV-DNA, INHA, and EBV-VCA IgA + INHA had low diagnostic efficacy for Empty stagnate poisonously nasopharyngeal carcinoma and EBV-VCA IgA + INHBB + INHA had moderate diagnostic efficacy for Empty stagnate poisonously nasopharyngeal carcinoma. The AUC of EBV-DNA, EBV-VCA IgA, INHBB, INHA, EBV-VCA IgA + INHBB + INHA, EBV-VCA IgA + INHA for Phlegm-type condensation nasopharyngeal carcinoma were 0.589, 0.514, 0.519, 0.695, 0.674, 0.683, and the AUC of INHA, EBV-VCA IgA + INHBB + INHA and EBV-VCA IgA + INHA was > 0.5 and *P* < 0.05, so INHA, EBV-VCA IgA + INHBB + INHA and EBV-VCA IgA + INHA had lower diagnostic efficacy for Phlegm-type condensation nasopharyngeal carcinoma. The AUC of EBV-DNA, EBV-VCA IgA, INHBB, INHA, EBV-VCA IgA + INHBB + INHA, and EBV-VCA IgA + INHA for Blood-type condensation nasopharyngeal carcinoma were 0.579, 0.524, 0.529, 0.587, 0.547, and 0.525, respectively, but the *P* > .05, so the above indexes do not have diagnostic efficacy for Blood-type condensation nasopharyngeal carcinoma (Fig. 4).

## 4. Discussion

NPC is a malignant tumor of the head and neck with a high incidence in the southern and southeastern coastal areas of China. Approximately 80% of NPC cases worldwide occur in China.^[19]^ Because of its atypical clinical symptoms and the concealment of nasopharyngeal anatomy, most patients with nasopharyngeal carcinoma have advanced stage and distant metastasis at the time of diagnosis.^[20]^ Although various imaging examinations, such as CT and MRI, can locate the diagnosis and clinical staging of nasopharyngeal carcinoma, they are not sensitive enough to the early stage, curative effect judgment, and late stage evaluation of the disease. In recent years, with the deepening of research on tumor markers, Inhibin B has gradually been considered a valuable biomarker in various cancer types.^[21]^ This study focused on Inhibin B expression in 35 patients with nasopharyngeal carcinoma. It was found that Inhibin B expression was lower in nasopharyngeal carcinoma compared with adjacent tissues, and its expression was related to clinical stage, lymphatic metastasis, and TCM syndromes, but not to T stage, sex, and age, suggesting that Inhibin B may be involved in the disease progression of nasopharyngeal carcinoma, and its low expression may be related to the deterioration of the disease. The expression of INHA is lower than that of INHBB, and the positive rates of INHA and INHBB in patients with stage III and IV are lower than those in patients with stages I and II, and the positive rates of INHA and INHBB in patients with lymphatic metastasis were also significantly reduced, indicating that INHA can better indicate the progress of nasopharyngeal carcinoma than INHBB. One study found that the expression of INHBB was lower in 39 nasopharyngeal carcinomas than in 16 chronic nasopharyngitis tissues, and the expression of INHBB was correlated with the clinical stage of lymphatic metastasis, while there was no clear correlation between gender and age, as well as T stage.^[22]^ EBV-DNA concentrations in nasopharyngeal cancer patients reflect the tumor load, and patients with higher EBV-DNA concentrations are more likely to develop distant metastases within 1 year of treatment.^[23]^ Nonetheless, the prognostic significance of EBV-DNA in non-endemic areas remains to be explored due to differences in sample types, DNA isolation procedures, PCR assays performed at different institutions, and the lack of a global standard for detecting EBV-DNA.^[24]^ EB-VCA IgA, as a late-synthesized structural protein, is present in the nucleus and cytoplasm of the cell and can be present in the host body throughout its life, which can contribute to the early diagnosis of nasopharyngeal carcinoma,^[25]^ but its sensitivity and specificity are low, and its application in the screening of nasopharyngeal carcinoma in asymptomatic people is limited.^[26]^ Inhibin B, a member of the TGF-β superfamily, is expressed at a high level in the reproductive system and plays an important role in the development and pathological process of tumors such as colorectal carcinoma and renal clear cell carcinoma.^[27]^ This study aimed to improve the efficacy of combining Chinese and Western medicine in the diagnosis of nasopharyngeal carcinoma, while focusing on analyzing the relationship between EBV-DNA, EB-VCA IgA, INHA, and INHBB with clinical stage and Chinese medicine patterns in 134 nasopharyngeal carcinoma patients. The results showed that the levels of EBV-DNA and VCA IgA in nasopharyngeal carcinoma patients were higher than those in healthy populations, and that patients with Stage III + IV were higher than those of patients with Stage I + II (*P* < .05). The levels of INHA and INHBB in nasopharyngeal cancer patients were significantly lower than those in the healthy population, and the levels in patients with stage III + IV were lower than those in patients with stage I + II (*P* < .05), which indicated that the expression levels of serum INHA and INHBB were correlated with the TNM clinical stage and the occurrence of distant metastasis in patients with nasopharyngeal cancer. Chinese medicine diagnosis mainly regulates the state and function of the organism systematically from a macroscopic point of view, with remarkable efficacy, but there is a certain degree of subjectivity; therefore, it is particularly important to find an objective basis for Chinese medicine diagnosis and typing of nasopharyngeal carcinoma, in order to explore the relationship between Chinese medicine diagnosis and clinical staging and degree of differentiation.^[28]^ In this study, we found that there was no correlation between the EBV-DNA and VCA IgA levels of different TCM evidence types, and the INHA levels of patients with Phlegm-type condensation and Blood-type condensation were higher than those of Empty stagnate poisonously, while the INHBB levels of patients with empty stagnate poisonously, Phlegm-type condensation, and Blood-type condensation were higher than those of the burning sleepy knot, which suggests that burning sleepy knot with a more solid evil Qi and Empty stagnate poisonously would seriously affect the patients’ prognosis.

In our study, we found that the expression difference between clinical stages and TCM patterns of Inhibin B played an important role in predicting the degree of malignancy of nasopharyngeal carcinoma and evaluating the therapeutic efficacy, and the ROC analysis showed that the diagnostic efficacy of EBV-DNA was the best for nasopharyngeal carcinoma among the single assays. Compared with the single EBV-VCA IgA antibody test, the combination of EBV-VCA IgA and Inhibin B (INHA, INHBB) can improve the diagnostic value of nasopharyngeal carcinoma, which can help to provide a basis for the diagnosis of nasopharyngeal carcinoma in terms of staging, diagnosis, and individualized treatment plan. Among the TCM patterns, EBV-DNA and INHA have low diagnostic efficacy for empty stagnate poisonously, while EBV-VCA IgA combined with INHA can improve the diagnostic efficacy of empty stagnate poisonously, and the combination of EBV-VCA IgA, INHBB, and INHA has medium diagnostic efficacy for empty stagnate poisonously, INHA,EBV-VCA IgA + INHBB + INHA, and EBV-VCA IgA + INHA have low diagnostic efficacy for Phlegm-type condensation nasopharyngeal carcinoma, which can further provide the basis for the diagnosis of nasopharyngeal cancer, diagnosis of the disease, and individualized treatment plan.

There are limitations to this study, such as the small specimen size, and a large number of clinical specimens are needed to verify the results. It is hoped that a large multicenter prospective study will be conducted in the future to further clarify the value of Inhibin B in the diagnosis of nasopharyngeal carcinoma and the assessment of TCM patterns to provide an objective basis for the prognosis of nasopharyngeal carcinoma.

## 5. Conclusions

The expression of Inhibin B may be related to the progression of nasopharyngeal carcinoma, and it has certain typing significance for different TCM syndromes of nasopharyngeal carcinoma, which is helpful for TCM typing diagnosis.

## Author contributions

**Conceptualization:** Nuoya Ma, Xin Deng, Fei Xu, Guoying Zou.

**Data curation:** Nuoya Ma, Qing Liu, Qi Guo.

**Writing – original draft:** Nuoya Ma, Xin Deng.

**Methodology:** Kun Yan, Yan Yang, Fei Xu, Guoying Zou.

**Project administration:** Fei Xu, Guoying Zou.

**Writing – review & editing:** Fei Xu, Guoying Zou.

**Funding acquisition:** Guoying Zou.
